# Evaluation of the Acceptability and Feasibility of the Social Attention and Communication Surveillance-Revised (SACS-R) Tool for Early Identification of Autism in Preterm Infants: The Identify and Act Study

**DOI:** 10.3390/children12091130

**Published:** 2025-08-27

**Authors:** Gayatri Athalye-Jape, Sarah Pillar, Sudharshana Saminathan, Kexian Wu, Stephanie Sherrard, Emma Dudman, Mary Sharp

**Affiliations:** 1 Neonatology Directorate and Neonatal Follow-Up Program, King Edward Memorial Hospital, Perth, WA 6008, Australia; 22828991@student.uwa.edu.au (S.S.); emma.dudman@health.wa.gov.au (E.D.); mary.sharp@health.wa.gov.au (M.S.); 2 Neonatal Health Team, Early Life and Life-Course Health, The Kids Research Institute, Perth, WA 6009, Australia; 3 School of Medicine, University of Western Australia, Perth, WA 6009, Australia; stephanie.sherrard@health.wa.gov.au (S.S.); 22810559@student.uwa.edu.au (K.W.); 4 CliniKids, The Kids Research Institute Australia, Perth, WA 6008, Australia; sarah.pillar@thekids.org.au

**Keywords:** preterm, autism, social attention and communication, quality improvement

## Abstract

**Highlights:**

**What are the main findings?**

- Routine use of the SACS-R tool in a high-risk preterm infant follow-up clinic was found to be both feasible and acceptable to caregivers and clinicians, with strong support for its integration into standard practice.
- The study identified 8.5% of screened children as having a high likelihood of autism—consistent with existing prevalence data—and highlighted delays in pointing and imitation as key early markers in this population.

**What is the implication of the main finding?**

- The successful implementation of the SACS-R tool in a high-risk preterm clinic suggests it can be effectively integrated into routine developmental surveillance, enabling earlier identification of autism in vulnerable populations.
- Early detection facilitates timely referral to intervention services, potentially improving long-term developmental outcomes for preterm infants.

**Abstract:**

Introduction: Preterm birth is associated with a 3.3-fold increased likelihood of autism diagnosis, with lower gestational age conferring higher likelihood. In Australia, autism is typically diagnosed at around age four, potentially missing the optimal neuroplasticity window before age two. The Social Attention and Communication Surveillance—Revised (SACS-R) tool identifies early autism signs in children aged 11–30 months, enabling pre-emptive intervention. Aims: This quality improvement (QI) study assessed the acceptability, and feasibility of SACS-R for early detection of autism traits in 12-month-old infants born very preterm/VP (gestation < 32 weeks), from both caregiver and clinician perspectives. Methods: From September 2024 to February 2025, 47 VP infants attending the 12-month Neonatal Follow-up Clinic (NNFU) at King Edward Memorial Hospital (KEMH), Western Australia, were assessed using SACS-R. Caregivers completed acceptability and feasibility questionnaires; clinicians completed similar surveys. Forty-seven infants met inclusion criteria; 12 clinicians provided responses. Results: Of 47 infants, 4 (8.5%) were identified as having a high likelihood of autism and referred for early intervention. Among caregivers, 29 (61%) provided complete acceptability responses and 28 (59%) feasibility responses, both predominantly positive. Clinicians reported high satisfaction (83%) and ease of use (91%), with 74% supporting routine implementation. Concerns included parental understanding and overlap with other assessments. Conclusions: Our QI study indicates that the SACS-R is highly acceptable and feasible in neonatal follow-up for preterm infants. Larger-scale evaluation of diagnostic accuracy and practical refinements based on feedback are warranted to support routine integration in early surveillance programs.

## 1. Introduction

Autism spectrum disorder (ASD; hereafter ‘autism’) is a neurodevelopmental condition that affects social communication, information processing and behaviors [1]. The number of Australians diagnosed with autism has surged in recent years, increasing from 1 in 125 in 2018 to 1 in 91 in 2022 with prevalence continuing to rise [2].

The odds of autism in preterm-born children are 3.3 times higher than in the general population [3], with studies quoting increasing prevalence with decreasing gestational age; 6.1% (22–27 weeks), 2.6% (28–33 weeks), and 1.9% (34–36 weeks) [3]. A recent meta-analysis reported pooled prevalence rates of 2.8% in very preterm and 2.1% in overall preterm populations [4]. The disruption of structural and functional neural pathways impacting on social interaction, communication and behavior, neuroinflammation, and perinatal complications (such as growth restriction, sepsis, intraventricular hemorrhage, and chronic lung disease) in addition to genetic and environmental predisposition may contribute to this significantly increased likelihood of autism diagnosis [5,6].

The current mean age of autism diagnosis is 4.1 years or even older age [7,8], despite research showing the ability to predict autism diagnosis using early behavioral markers in infancy [9]. Early intervention (EI) for children with autism aims to nurture individual strengths, reduce challenges associated with differences in social communication, language development, sensory processing, adaptive and learning skills, and provide appropriate family support. The greatest shifts in developmental trajectory are seen when EI is employed during the period of greatest neuroplasticity, particularly in the first 1000 days of life [10,11]. Despite clear evidence that autism diagnosis can be predicted by behavioral markers in infancy [9], many Australian children miss out on accessing appropriate EI supports during this critical window, which can have a cascading effect on later development [12].

Validated developmental surveillance tools are essential for identifying infants with a high likelihood of autism. While the M-CHAT and M-CHAT-R/F are commonly used between 16 and 30 months, their diagnostic accuracy is variable, especially in low-risk populations and at 18 months. In contrast, the Social Attention and Communication Surveillance—Revised (SACS-R) checklist, developed in Australia, shows promising psychometric properties and can be used from 12 months of age. It has demonstrated high PPV (81–83%), NPV (99%), specificity (99–99.53%), sensitivity (82–84%), and diagnostic stability (88%) at 24 months [13,14,15,16].

The Social Attention and Communication Surveillance—Revised (SACS-R) checklist seems to be a promising autism screener. The ability to use the screener on infants as young as 12 months allows for potential identification during the critical window for targeted identification and referral to EI. Despite its strengths, the SACS-R has not been routinely used in very preterm (VP) infants, a high-likelihood group for autism diagnosis [17,18,19]. In our previous work, we retrospectively reviewed a small cohort of VP children in our neonatal follow-up (NNFU) program who had undergone SACS-R screening at 24 months based on clinician suspicion or parental concern. Of the 12 children screened, 11 were later diagnosed with autism, yielding a diagnostic accuracy of 91.7% and a median time to diagnosis of 11 months. Screening took approximately 10 min and did not prolong clinic visits [20].

Building on these promising retrospective findings, the current study aimed to prospectively assess the feasibility and acceptability of routine SACS-R implementation in VP infants within our NNFU program. Using quality improvement (QI) methodology, we gathered perspectives from both caregivers and clinicians to inform future integration of early autism surveillance in high-risk neonatal populations.

## 2. Materials and Methods

### 2.1. Study Design and Objectives

We conducted this study as a six-month quality improvement (QI) cycle using the Plan–Do–Study–Act (PDSA) framework [21]. The identified gap was the absence of evidence regarding the use of the SACS-R tool in VP infants despite their elevated likelihood of autism diagnosis. Our approach involved planning and implementing the SACS-R tool within a neonatal follow-up context, collecting feedback, and analyzing results to inform future iterations. Our goal was to refine processes for routine implementation and to establish a clinical pathway for early autism identification and referral to EI in this high-likelihood group. Our SMART (Specific, Measurable, Achievable, Relevant, Time-Bound) objectives were to screen at least 90% of children enrolled in the NNFU at their 12-month visit using the SACS-R checklist within 6 months of project initiation and to refer 100% of children who screened positive for early intervention.

We utilized a cross-sectional observational design comprising three components: (a) administration of the paper-based 12-month SACS-R checklist during routine clinical appointments, (b) caregiver-completed surveys assessing acceptability and feasibility of the tool (Appendix A), and (c) clinician-completed post-study surveys assessing their experiences with acceptability and feasibility (Appendix A).

Local institutional governance was obtained prior to commencing the study (GEKO number: 54681).

### 2.2. Setting and Participants

Participants were recruited between 2 September 2024 and 28 February 2025 from the Neonatal Follow-Up Unit (NNFU) at King Edward Memorial Hospital (KEMH), Perth, Western Australia. Participation was voluntary; data were de-identified during analysis. Completion of caregiver questionnaires implied informed consent. Clinician responses were anonymous, and no remuneration was provided.

#### 2.2.1. Infants

Inclusion criteria: VP infants discharged after neonatal intensive care stay from KEMH and aged between 12 and 16 months corrected gestational age (CGA) at the assessment.

Exclusion criteria: Infants born >32 weeks gestation and discharged from KEMH NICU who were eligible for assessment during the QI period.

#### 2.2.2. Clinicians

Inclusion criteria: Clinicians were eligible if they conducted at least one follow-up consultation (Griffiths-III assessment) during the study period. All 12 participating clinicians were either neonatologists/neonatal trainees or developmental pediatricians actively involved in routine follow-up care.

Exclusion criteria: If not eligible as above.

### 2.3. Tools Used

The following were used during assessments

1. Demographic questionnaire: Completed by clinicians, and included patient gestation at birth (GA), birth weight (BW), significant complications during NICU stay, ethnicity, languages spoken at home, primary language, parental education, family history of autism, maternal comorbidities during antenatal period and reported developmental concerns.
2. SACS-R 12-month checklist: The SACS-R evaluates early social-communication behaviors across five key items (eye contact, pointing, gestures, imitation, and response to name) and five non-key items (social smiling, babble, following a point, early word use, comprehension). Each behavior is coded as ‘typical’ (eye contact: child makes regular and consistent eye contact with examiner and caregiver across different settings, pointing: child points to show something that is out of reach and turns to look at you, gestures: child uses gestures such as waving or clapping, imitation: child copies examiner’s or caregiver’s actions, response to name: child consistently responds to his/her name when called) or atypical; ≥3 atypical key items classify a child as ‘high likelihood (HL)’ for autism, prompting referral to early intervention if available (Appendix A).
3. Caregiver acceptability and feasibility questionnaires: Acceptability (8 items) and feasibility (11 items) were rated on a 5-point Likert scale with open-ended questions regarding tool clarity, relevance, mode of administration, and emotional impact (Appendix A).
4. Clinician acceptability and feasibility questionnaires: Administered post-study via REDCap, using similar 5-point scales and open-ended questions regarding ease of use, practicality, and integration into clinical workflow (Appendix A).

### 2.4. Procedures and EI Referral

Eligible caregivers were approached during clinic check-in and provided with study information. Informed consent was implied by voluntary questionnaire completion. During the consultation, clinicians completed the demographic form and SACS-R checklist as part of routine developmental surveillance. Caregivers completed feedback surveys during or immediately after the appointment. De-identified data were entered into REDCap for analysis. Following completion of the study period, clinicians were emailed the acceptability and feasibility surveys.

Infants identified as ‘HL’ for autism based on SACS-R scoring were referred to an evidence-based local EI program for autism-specific support and flagged for ongoing developmental monitoring. They will be referred for confirmation of an autism diagnosis if symptoms persist despite EI.

### 2.5. Data Analysis

Data were analyzed using REDCap. Descriptive statistics (frequency, percentage) were calculated for demographic variables, clinician and caregiver responses, and SACS-R outcomes. SACS-R checklist scoring focused on the number of atypical responses, particularly across key items. Comparisons were made between participants with complete and incomplete caregiver data to assess potential response bias. Open-ended responses were thematically analyzed to identify common concerns and suggestions regarding tool feasibility and acceptability. Three independent reviewers (GAJ, Su Sa, and KW) manually coded the responses to identify recurring patterns and themes. Discrepancies in coding were discussed among all authors and resolved through consensus.

## 3. Results

**Figure 1:**
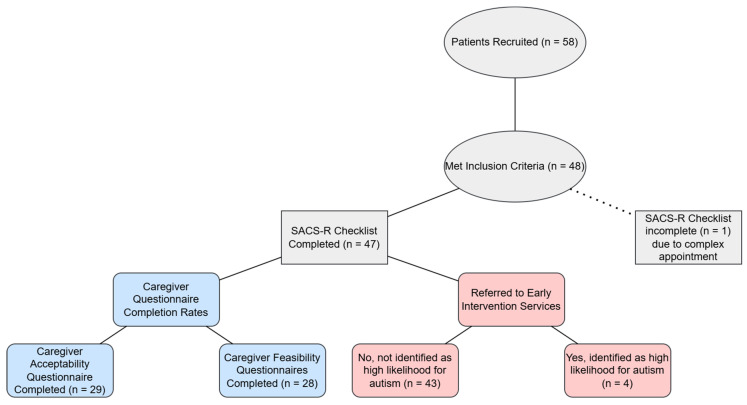
Flowchart showing details of participant recruitment.

From a total of 58 parents/families who attended NNFU during the study period, 48 met the inclusion criteria (Figure 1). One family was excluded due to a partially completed SACS-R checklist, with appointment complexity cited as the reason for non-completion. A total of 47 children were included.

### 3.1. Demographic Characteristics

The median (IQR) GA and BW was 29.0 (28–30) weeks and 1099 (883–1400) grams, with a median age at assessment of 13.5 (12–15) months. Gender distribution of males (n = 22) and females (n = 25) was almost equal for the 47 eligible children. Of the 47 children’s families, only 25 (53.2%) had their demographic questionnaires completed due to inadequate time, complex assessments, and child fatigue necessitating appointment termination. Table 1 highlights the demographic characteristics of the 25 families with majority being from Caucasian ethnicity (44%), with most having completed tertiary education (64% for both parents) and English as the primary language spoken at home (60%). A sibling with autism was reported by one family (2%), and family history of autism was reported by 40%.

### 3.2. Screening Using the 12 Month-SACS-R Checklist

We screened all VP-born children (100%) who attended their appointment at their 12-month corrected age visit using the SACS-R checklist and referred 100% of those who screened ‘HL’ (4/47) for EI. Of the key items being measured in the SACS-R tool, ‘pointing’ was the most frequent item that was deemed ‘atypical’ (14/47; 30%) followed by ‘imitation’ (7/47; 15%), ‘gestures’, and ‘response to name’ (5/47 each; 10.6%). All four children referred to EI had these key items deemed as ‘atypical.’

Among non-key items, ‘speaks 1–3 words’ and ‘understands simple instructions’ were most frequently ‘atypical’ (9/47 each; 19%), followed by ‘conversational babble’ (7/47; 15%), ‘follows point’ (4/47; 8.5%), and ‘social smile’ (1/47; 2%).

### 3.3. Caregiver Acceptability and Feasibility

Regarding caregiver questionnaire completion rates, 29 (62%) parents completed the acceptability, and 28 (60%) completed the feasibility questionnaire. Only fully completed questionnaires were included in the analysis. The commonest reason for non-completion was time constraints or appointment complexity including an un-cooperative or challenging to assess child (fatigue, hunger, nap time, etc.).

**Figure 2:**
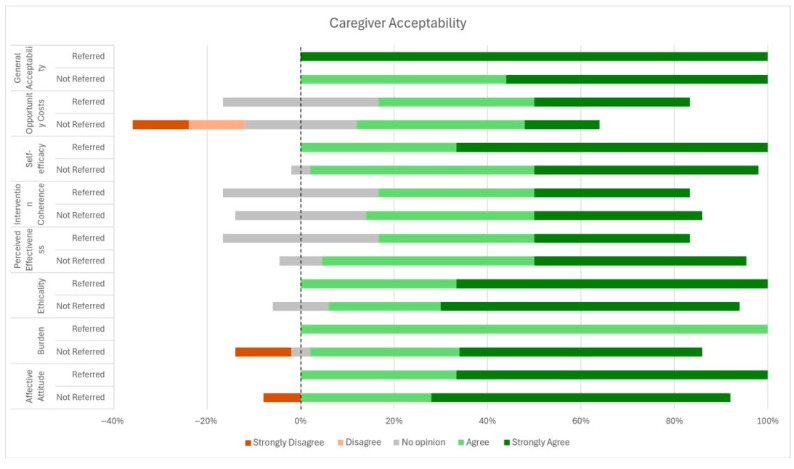
Caregiver acceptability of the SACS-R tool.

Overall, there was a high level of acceptability and positive perception of the SACS-R tool (Figure 2), with all participants rating general acceptability positively, and the majority of participants positively rating acceptability across domains. Affective attitude, ethicality, perceived effectiveness, intervention coherence and self-efficacy all were positively received (100%). A minority (3/29, 10.3%) reported that the burden of completing the SACS-R tool required huge effort, but notably all respondents with this view were carers of children not referred for EI. Opportunity cost was the only item out of the entire form that yielded a notable proportion of negative responses, with 20.7% (n = 6) parents reporting ‘agree or strongly agree’ that completing the SACS-R tool prevented them from engaging in other activities they would have valued more.

**Figure 3:**
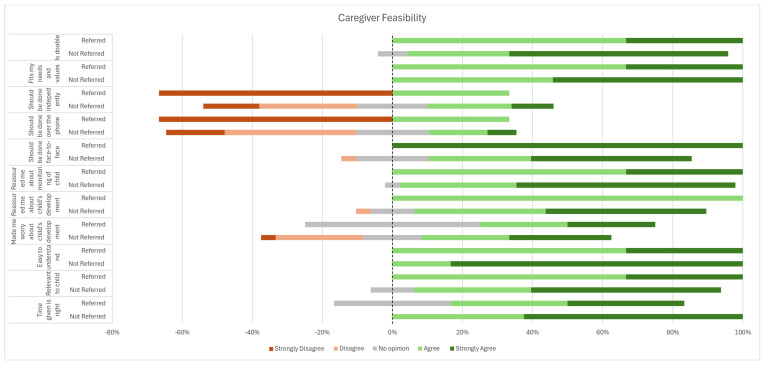
Caregiver feasibility of the SACS-R tool.

A similar distribution was observed for the caregiver feasibility questionnaire (Figure 3), with an overall positive response across feasibility domains of time, relevance, clarity, affect (worry or reassurance about child development), modality (face-to-face completion), and manner. A high dispersion was observed in caregiver responses to the question regarding whether the SACS-R tool raised concerns about the child’s development (Affect 1). No caregivers of children referred to EI answered ‘agree or strongly agree’ for this question.

Notably, lower GA appeared to be associated with a slightly higher perceived burden. Among parents of children born ≤ 28 weeks, the majority rated the effort of completing the SACS-R tool as needing ‘a little effort’ (n = 10, 71.4%), while a small proportion reported needing ‘no effort at all’ (n = 2, 14.3%). In contrast, among those born between 28 and 31 weeks, most parents rated the task as requiring ‘no effort at all’ (n = 10, 73%), with fewer selecting ‘a little effort’ (n = 2, 13.3%). Moreover, lower GA also appeared to be associated with a stronger preference for face-to-face delivery of the SACS-R. Among parents of children born ≤ 28 weeks, 61.5% (n = 8) strongly agreed that the tool should be completed in person with a clinician, while only 46.7% (n = 7) of those whose children were born between 28 and 31 weeks expressed the same level of agreement.

### 3.4. Clinician Characteristics

All 12 clinicians who assessed patients during the study period completed the post-study questionnaires, representing a 100% response rate. The clinician cohort included both developmental pediatricians (n = 2), senior neonatal trainees (n = 5), and neonatologists (n = 5). There was notable variation in patient load, with the number of patients assessed per clinician over the six-month period ranging from 2 to 17. Additionally, 50% of the clinicians had working experience of seven or more years in developmental assessments of high-risk children; the remainder 50% were mainly trainees/junior consultants and had variable experience between 1 and 3 years. Of the twelve clinicians who assessed patients, seven received formal external training in SACS-R administration, whereas the remaining five were internally trained by the neonatologists. Importantly, all clinicians (100%) reported administering the SACS-R checklist during every eligible consultation.

### 3.5. Clinician Acceptability and Feasibility

**Figure 4:**
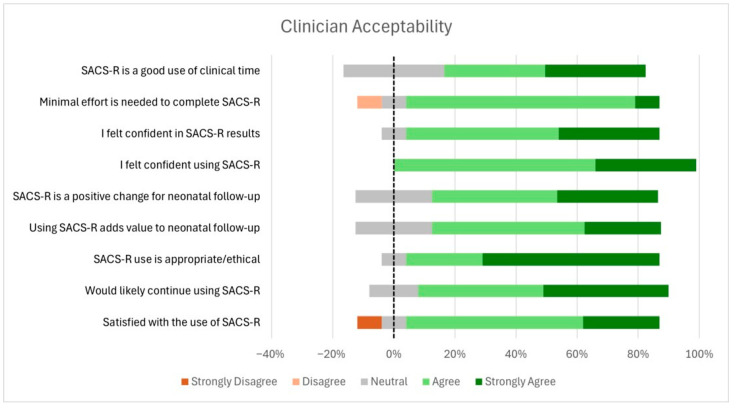
Clinician acceptability of the SACS-R tool.

Due to the use of a modified Likert scale with variable response options across items, clinician acceptability results are presented in both bar graph format (Figure 4) and tabular format (Table 2) to enhance clarity and interpretability.

In terms of acceptability, clinicians reported a generally high level of satisfaction, with a majority (83%) reporting either satisfaction (58%) or high satisfaction (25%). Most clinicians (82%) stated they were ‘likely or very likely’ to continue using the SACS-R tool, and 92% agreed that its use was both appropriate and ethical in the neonatal follow-up setting (Table 2). While 75% of clinicians agreed that the SACS-R tool enhanced the value of neonatal follow-up and represented a positive change in clinical practice, 25% remained neutral on this point. In terms of perceived effort, the tool was generally seen as manageable: 83% reported requiring little to no effort to complete it, while only 8% felt that it demanded significant effort. Finally, 66% of clinicians agreed that completing the SACS-R tool was a worthwhile use of clinical time. However, 33% were neutral on this, citing redundancy with existing assessments.

**Figure 5:**
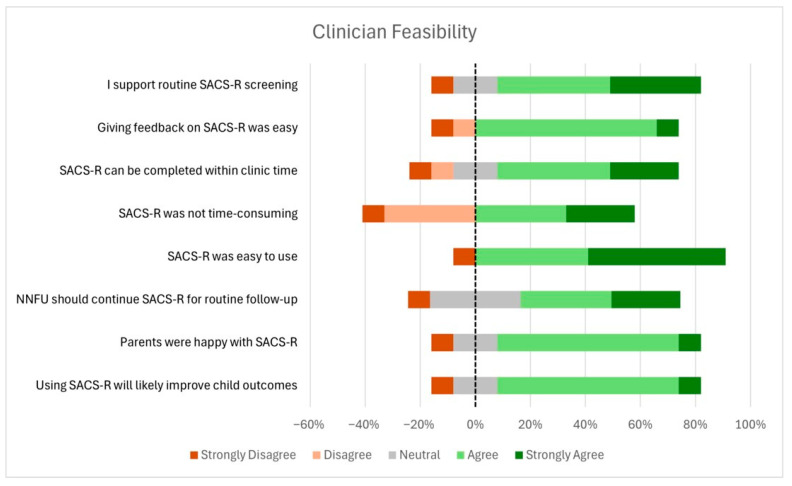
Clinician feasibility results.

Clinician feasibility was evaluated across four domains: demand, implementation, practicality, and adaptation, using both quantitative responses and open-ended feedback to better understand their experience using the SACS-R tool. The quantitative responses are represented in Figure 5, with the majority of clinicians reporting a high level of feasibility across domains.

In terms of demand, 74% of clinicians either agreed or strongly agreed that the use of the SACS-R tool could positively influence a child’s developmental trajectory, and that parents appeared receptive to the tool. However, 16% were neutral on this point and 8% strongly disagreed. Similarly, only 58% of clinicians agreed that the NNFU should continue using the SACS-R tool for routine follow-up, with 33% remaining neutral and 8% disagreeing.

Implementation was largely viewed favorably, with 92% of clinicians agreeing that the SACS-R tool was easy to use. However, 41% still considered the tool time-consuming, while 33% remained neutral, highlighting that ease of use does not necessarily equate to efficiency in time-pressured settings.

In the domain of practicality, 16% of clinicians reported that the tool could not be completed in a timely manner during the allocated clinic visit (within 60 min appointment time with a Griffiths-III and medical assessment), and another 16% were neutral. Additionally, 16% noted that giving feedback based on the SACS-R tool was not always straightforward, perhaps suggesting limitations in how seamlessly the tool fits into routine clinical processes.

When it came to adaptation, 74% of clinicians supported the ongoing use of the SACS-R tool as a routine part of neonatal follow-up screening. However, 16% were neutral, and 8% disagreed, with some citing overlap with existing tools as a reason for hesitancy.

Open-ended feedback provided by clinicians across the forms have been collated in Table 3 and Table 4.

As represented in Table 3, multiple clinicians strongly supported the use of autism screening in neonatal follow-up, noting its value in enhancing early autism detection and access to intervention. While some raised questions about validation of the SACS-R specifically, which highlights an important knowledge gap for additional staff training, all agreed on the importance of autism screening. One clinician also noted the tool was acceptable to parents.

As represented in Table 4, clinician feedback highlighted some potential concerns regarding the implementation of the SACS-R tool. Key issues included demands on both clinician and parent time, as well as gaps in information for the parent about the tool and the communication of results. Clinicians offered insightful and practical suggestions to overcome the issues they perceived, including suggestions to shorten the parent demographic questionnaire, sending out questionnaires in advance of assessments to reduce the time pressure within assessments, and the potential added value to providing additional resources for parents to support their understanding of the tool and the results. Some clinicians also noted overlap with existing developmental assessments (Griffiths-III) and questioned the tool’s added clinical value, indicating that the unique purpose of the SACS-R tool may not have been effectively communicated to participating clinicians.

## 4. Discussion

This is the first West Australian study to evaluate caregiver and clinician acceptability and feasibility of implementing the SACS-R tool for identifying autism in a high-risk preterm infant follow-up clinic. Findings from our QI pilot study suggest that routine administration of the SACS-R during the 12-month follow-up appointments was both feasible and acceptable from both the caregiver and clinician perspectives.

In total, 8.5% (4/47) of children screened were identified as having a high likelihood of developing autism and were subsequently referred to early intervention services. This aligns with the existing literature that 7–8% of preterm children would likely be diagnosed with autism [22,23,24], compared to a rate of 2–3% in the general population [2,25]. All referred children at a corrected age of 13.5 (12–15) months, demonstrated atypical behaviors in both ‘pointing’ and ‘imitation,’ two key items of the SACS-R. Notably, absence of ‘pointing’ was the most frequently observed atypical trait overall, consistent with literature where pointing gestures may appear at 12–15 months corrected age in preterm infants [26]. Pointing delay may be linked to challenges in joint attention, motor coordination and social-cognitive development [27]. Suttora et al. reported that in preterm children, producing pointing gestures at 12 months was positively linked to vocabulary development by 24 months, while combining gestures with words at 18 months was associated with stronger morphosyntactic abilities at 24 months [26]. Low rates of pointing in 18-month-olds with increased likelihood for autism diagnosis, such as those with a sibling with an autism diagnosis or extremely preterm-born infants, have been reported previously [27]. The emergence of ‘imitation’ may also be delayed in preterm infants often occurring closer to 9–12 months corrected age [28,29] thus indicating ‘real’ delay in imitation in our cohort.

This QI pilot study was the first of its kind in Western Australia to assess the feasibility and acceptability of implementing the SACS-R tool for early autism detection in a high-risk preterm infant follow-up clinic, filling a critical gap in regional research. The design effectively captured both caregiver and clinician perspectives, revealing high levels of satisfaction and confidence in using the tool. The alignment of autism detection rates with existing literature reinforced the tool’s validity, while the identification of specific atypical behaviors, particularly delays in pointing and imitation, perhaps add valuable insight into early markers of autism in preterm populations. The study also highlights practical implementation strengths, such as clinician ease of use and caregiver preference for face-to-face administration, supporting the tool’s integration into routine clinical practice. Importantly, the findings justify further large-scale research and underscore the potential of SACS-R to enhance early developmental surveillance in vulnerable cohorts.

The study has several limitations include the small sample size (n = 47) limiting the generalizability of the results, particularly as this sample does not include those children who missed their appointments and may be more ‘at-risk’ [30,31,32]. Being a pilot QI initiative, our study was exploratory in nature and not designed to establish causality or long-term outcomes. Furthermore, this was a single-center study conducted in a high-risk preterm follow-up clinic, which may limit the applicability of findings to other clinical settings (community/child health nurses) or populations (moderate/late preterm and other ethnic groups including Aboriginal and Torres Strait Islander people). Furthermore only 53% of families provided demographic data, which limited the interpretation of background variables.

Our study analysis was primarily descriptive, which was appropriate given its pilot nature and focus on feasibility and acceptability. However, we acknowledge that the absence of basic comparative analyses, such as stratification by gestational age, limits the depth of interpretation. Future studies with larger sample sizes and sufficient statistical power should incorporate such analyses to enhance the robustness and clinical relevance of the findings.

Caregiver responses to the SACS-R tool varied, although majority expressed feeling reassured and supported by ongoing surveillance. Additionally, caregivers expressed a clear preference for completing the SACS-R tool in face-to-face clinical settings. Phone-based administration was rated least favorably. These findings support the current model of incorporating screening within the clinic and resorting to telehealth in alternative circumstances (e.g., difficulty travelling or pandemic lockdowns). However, any alternative administration method must account for the complexity of the tool, which may require guidance from trained clinicians to ensure accurate responses.

Caregiver feedback also highlighted concerns around time burden and perceived ‘opportunity cost’ of participation, particularly in the context of high-risk follow-up where families are often navigating complex emotional and logistical challenges. While these concerns did not prevent engagement, they underscore the importance of designing screening to be minimally disruptive and sensitive to family needs. Strategies such as streamlining data collection, offering flexible participation options, and clearly communicating the potential benefits may help mitigate these concerns and enhance acceptability. These insights are valuable for informing the design of larger-scale studies and improving sustainability.

Clinicians noted perceived redundancy between the SACS-R and the Griffiths-III developmental assessments, which may have contributed to uncertainty regarding its unique clinical utility. Unlike standardized assessments that note whether children have met age-appropriate milestones, the SACS-R is designed to identify subtle, early markers of autism spectrum disorder and related social communication challenges, which may be present despite standardized developmental assessment scores falling within normal range. Emphasizing this distinction, along with its role in complementing rather than duplicating existing assessments, may improve clinician understanding and acceptance. Further training and integration into routine workflows could also help mitigate concerns and reinforce its value in early identification and referral pathways. Given the linguistic diversity within our sample, it is important to consider how non-English language backgrounds may influence the applicability and interpretation of the SACS-R tool. While the tool has demonstrated utility in various settings and is currently available in four languages—English, Spanish, Mandarin, and Slovak—its reliance on language-based social communication cues may still present challenges for families from culturally and linguistically diverse (CALD) backgrounds. This could affect both parental understanding of the tool and the accuracy of early social-communication assessments. Future research should explore further cultural adaptations and consider the inclusion of multilingual resources or interpreter support to enhance accessibility and equity in developmental surveillance.

Lastly, the study focused primarily on two developmental traits, pointing and imitation, being key items in the SACS-R, perhaps potentially overlooking the significance of other non-key items that may be relevant in preterm populations. Further research is warranted to explore the predictive utility of non-key items and their role in complementing key item marker-based screening.

## 5. Conclusions

In summary, we demonstrated that SACS-R could be implemented routinely for autism surveillance in our high-risk preterm NNFU clinic. Previous studies have reported similar findings but across community settings and only in term cohorts [33,34]. Importantly, our findings justify further large-scale research and underscore the potential of SACS-R to enhance early developmental surveillance in vulnerable cohorts.

## Figures and Tables

**Table 1:** Demographics of assessed families.

Demographics		Number of Patients
Sex	Male	11
	Female	14
Ethnicity	Aboriginal and Torres Strait Islander	4
	African	0
	Asian	2
	Caucasian	11
	Indian	6
	Maori	0
	Other	2
	Unknown	4
Developmental delay in child being assessed	Present	2
	Absent	20
	Unknown	3
Siblings with ASD	Present	1
	Absent	22
	Unknown	2
Family history of ASD	Present	10
	Absent	14
	Unknown	1
	Present; ADHD Only	5
	Present; ADHD and others	1
	Present; Speech and Learning Delays	2
	Absent	15
	Unknown	2
Language spoken at home	English Only	15
	English as the Primary Language + Additional Language(s)	4
	English as the Secondary Language + Additional Languages(s)	5
	Unknown	1
Maternal level of education	Primary	0
	Completed yr 10	2
	Completed yr 12	7
	Tertiary and above	16
Paternal level of education	Primary	1
	Completed yr 10	2
	Completed yr 12	6
	Tertiary and above	16
Maternal comorbidities ^@^	Diabetes	10
	Pre-eclampsia	4
	Chorioamnionitis	4
	Neurobehavioral Issues	5
	PPROM/oligohydramnios	2
	None	8

^@:^ multiple comorbidities were present in mother.

**Table 2:** Clinician Acceptability Results.

Clinician Acceptability [n (%)] n=12					
Were you satisfied with the use of SACS-R tool?	Very Unsatisfied	Unsatisfied	No Opinion	Satisfied	Very Satisfied
	1 (8)	0	1 (8)	7 (58)	3 (25)
Given the choice, would you be likely to continue using it?	Very Unlikely	Unlikely	No Opinion	Likely	Very Likely
	0	0	2 (16)	5 (41)	5 (41)
Do you think it is appropriate/ethical to use SACS-R tool? (n = 11)	Very Inappropriate	Inappropriate	No Opinion	Appropriate	Very Appropriate
	0	0	1 (8)	3 (25)	7 (58)
Using the SACS-R tool improves the value of neonatal follow-up:	Strongly Disagree	Disagree	Neutral	Agree	Strongly Agree
	0	0	3 (25)	6 (50)	3 (25)
Using the SACS-R tool is a positive change for neonatal follow-up	Strongly Disagree	Disagree	Neutral	Agree	Strongly Agree
	0	0	3 (25)	5 (41)	4 (33)
How confident did you feel using the SACS-R	Not at all	Slightly Confident	Neutral	Reasonably Confident	Very Confident
	0	0	0	8 (66)	4 (33)
I felt confident in the results of the SACS-R (n = 11)	Strongly Disagree	Disagree	Neutral	Agree	Strongly Agree
	0	0	1 (8)	6 (50)	4 (33)
How much effort did it take to complete the SACS-R tool?	No effort at all	A little effort	No Opinion	A lot of effort	Huge effort
	1 (8)	9 (75)	1 (8)	1 (8)	0
Completing the SACS-R is a worthwhile use of clinical time:	Strongly Disagree	Disagree	Neutral	Agree	Strongly Agree
	0	0	4 (33)	4 (33)	4 (33)

**Table 3:** Open-ended clinician feedback on best aspects of SACS-R tool.

Clinician Open-Ended Comments on the Best & Worst Issues about the SACS-R tool	Area of Feedback
Best—using an ASD screening tool is a really good idea for neonatal follow-up. I believe it will improve rates of kids accessing appropriate early intervention services	Value of ASD specific screening tool
I don’t know how well validated and sensitive this specific tool is, but I definitely think an ASD screening tool needs to be used at all points in Neonatal follow-up	
I can’t comment on this specific tool having value over other ASD screening tools; however, I do feel that adding a dedicated caregiver ASD screening tool increases the value of our follow-up	
Important Screening tool as not all parents may be aware of ASD signs	
I didn’t specifically ask if parents were “happy”, but completing the tool certainly seemed acceptable and reasonable to parents	Parent Feedback

**Table 4:** Open-ended clinician feedback on potential concerns about SACS-R tool.

Clinician Open-Ended Comments on the Best & Worst Issues about the SACS-R tool	Area of Feedback
I used SACS-R tool only couple of times that too in a busy clinic while dealing with complex patients so it’s difficult to comment	Challenging complex clinic environment
Related audit tools took some time but questionnaire quick	Length of questionnaires
I think the parents’ questionnaire could be shortened. It takes longer than the SACS-R	
Some assessments take much more time than others. It would probably be appropriate/ideal if this tool was sent out in advance along with the ASQ (and an appropriate accompanying explanation of what the tool was and how to complete it)	Ensuring parental understanding of tool
Worst issues—no accompanying explanation for parents, so relies on the clinician’s explanation. I had no opportunity to explore the process of addressing the issue further if the tool screens positive, hence can’t comment on this	
Should continue using this tool, but WITH some preamble text to explain purpose to parents	
The name of the tool & title on the top of the pages makes no mention of Autism—took some time to explain this and the value of early diagnosis & intervention—would be good if this was included	
None issues but all the ones I completely did not flag increased risk of ASD. If parents had no concerns themselves but the tool screened positive it may take some time to explain the results and address any questions	Complexity in results explanation
No worst issues but it will only be worthwhile if the best is shown to identify high risk patients in our setting and if we have someone to refer them to.	Sensitivity of SACS-R tool, Clarity about Follow-up Referrals
I don’t think it adds much to the clinical and developmental assessments.	Overlap with other developmental assessment tools
All items are already covered in Griffiths developmental assessment. Just need to get familiarity with the items on the checklist.	

**Figure A1:**
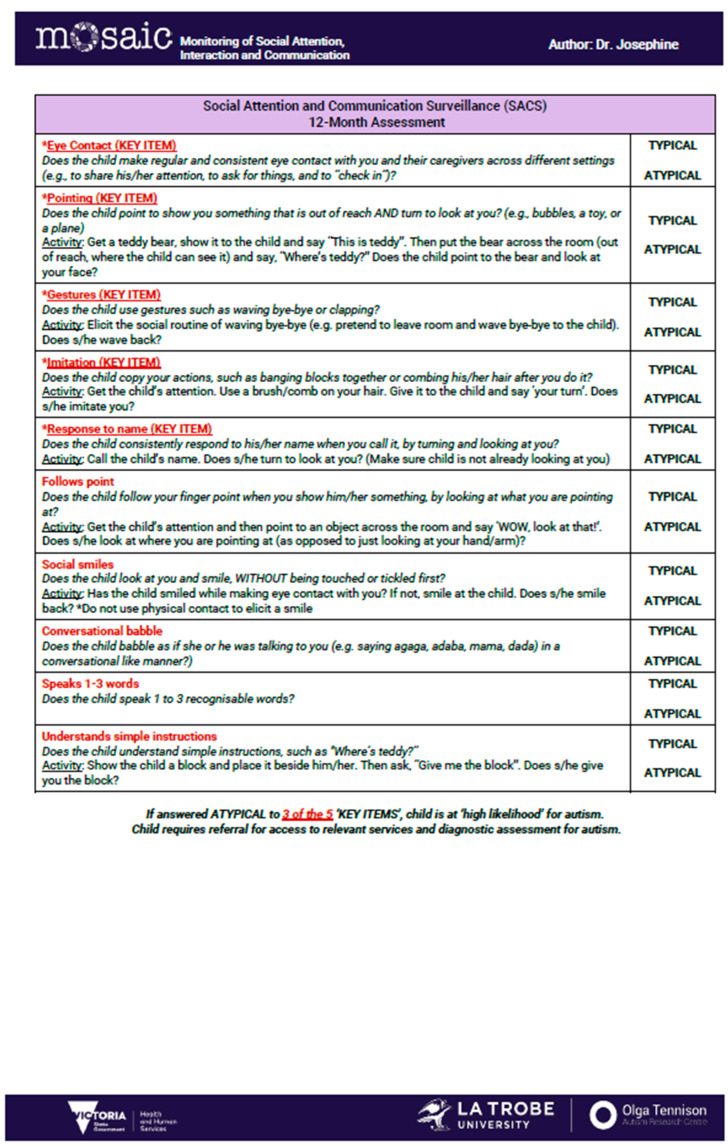
SACS-R checklist for 12-months.

**Figure A2:**
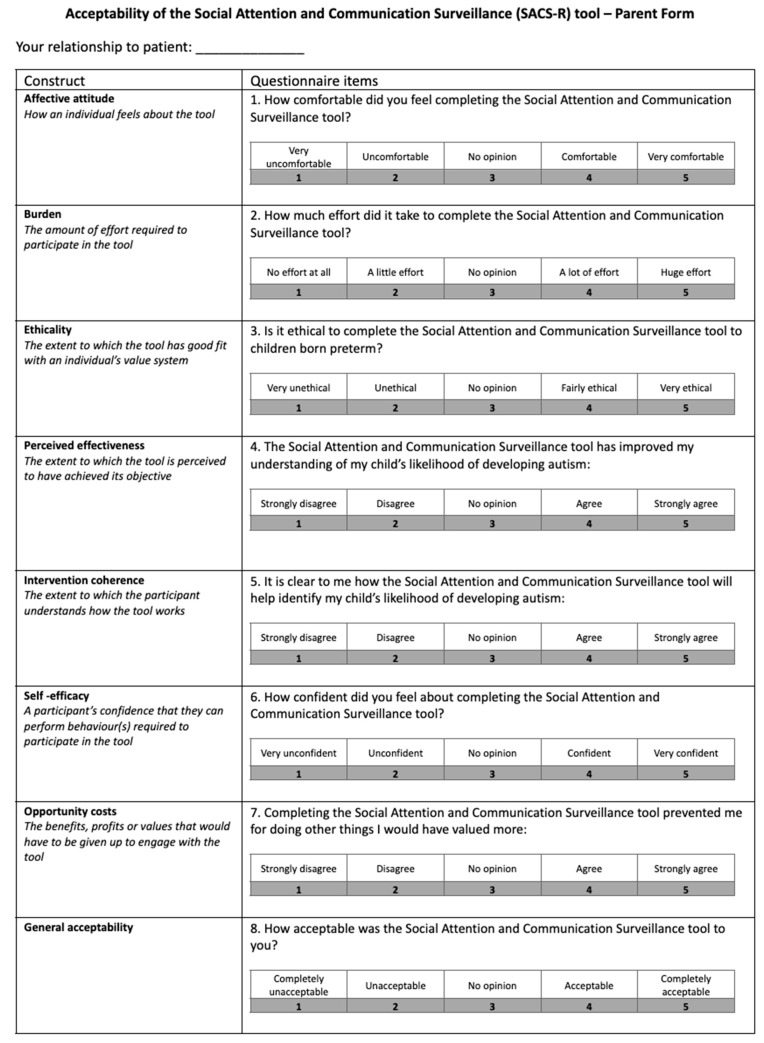
Acceptability of SACS-R for caregivers/ parents.

**Figure A3:**
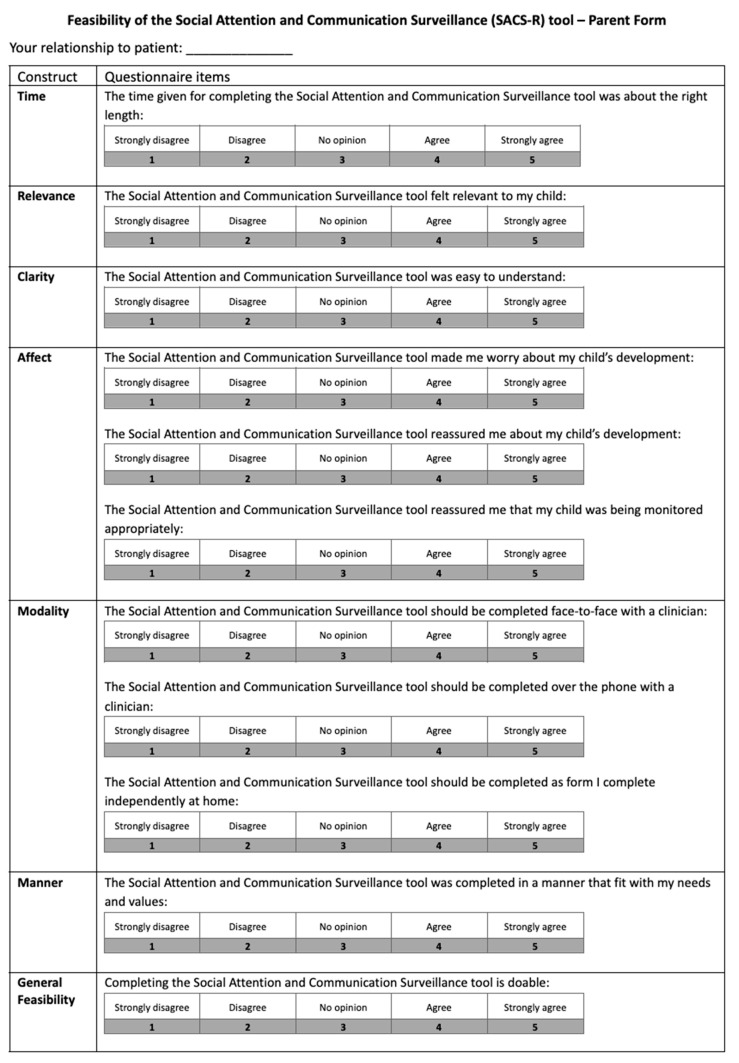
Feasibility of SACS-R for caregivers/ parents.

**Figure A4:**
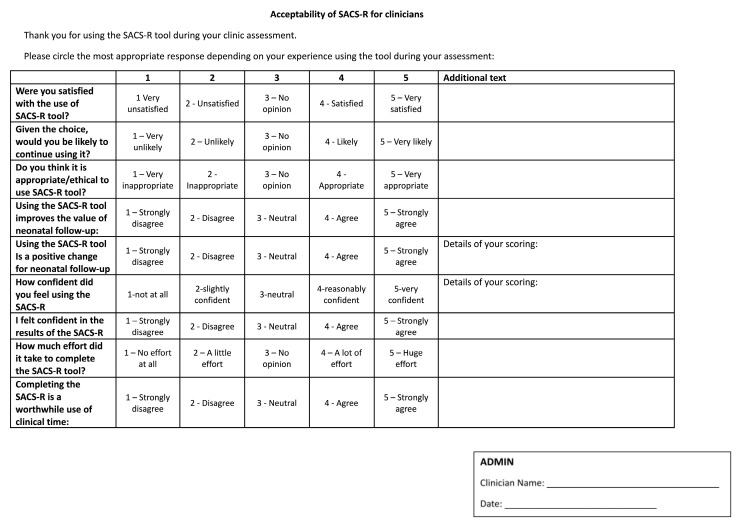
Acceptability of SACS-R for clinicians.

**Figure A5:**
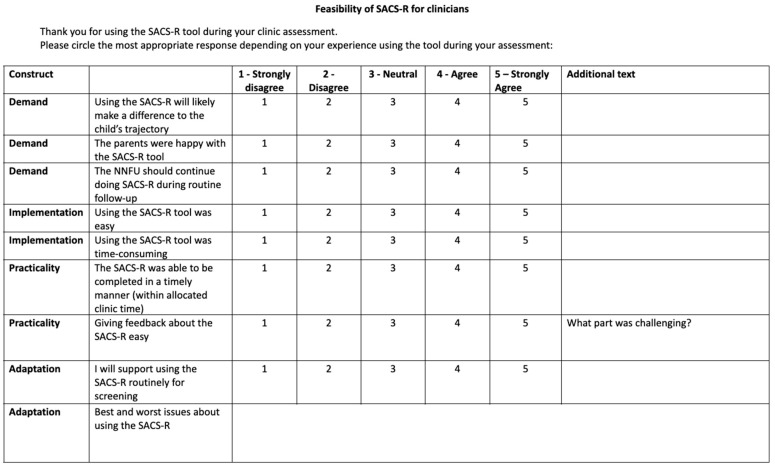
Feasibility of SACS-R for clinicians.

## Data Availability

Data can be requested by writing to the corresponding author.
